# Combined Detection of RUNX3 and EZH2 in Evaluating Efficacy of Neoadjuvant Therapy and Prognostic Value of Middle and Low Locally Advanced Rectal Cancer

**DOI:** 10.3389/fonc.2022.713335

**Published:** 2022-02-24

**Authors:** Likun Wang, Xueliang Wu, Wengui Xu, Lei Gao, Ximo Wang, Tian Li

**Affiliations:** ^1^ Department of Molecular Imaging and Nuclear Medicine, National Clinical Research Center for Cancer, Key Laboratory of Cancer Prevention and Therapy, Tianjin, Tianjin’s Clinical Research Center for Cancer, Tianjin Medical University Cancer Institute and Hospital, Tianjin, China; ^2^ Department of Gastrointestinal Surgery, Tianjin Medical University Nankai Hospital, Tianjin, China; ^3^ Department of General Surgery, First Affiliated Hospital of Hebei North University, Zhangjiakou, China; ^4^ Department of Ultrasound, Tianjin Medical University Nankai Hospital, Tianjin, China; ^5^ School of Basic Medicine, Fourth Military Medical University, Xi’an, China

**Keywords:** Runt-related transcription factor 3, histone-lysine N-methyltransferase EZH2, middle and low locally advanced rectal cancer, neoadjuvant therapy, prognosis, retrospective study

## Abstract

**Objective:**

This article investigated whether Runt-Related Transcription Factor 3 (RUNX3) and enhancer of zeste homolog 2 (EZH2) can be used to evaluate the clinical efficacy of neoadjuvant therapy and prognosis of locally advanced rectal cancer (LARC).

**Methods:**

Eighty LARC patients admitted to the Tianjin Medical University Cancer Institute/Hospital and First Affiliated Hospital of Hebei North University from Jan 2015 to Jan 2016 were enrolled. The patients were followed up for 60 months through hospital visits. All patients received neoadjuvant chemoradiotherapy (long range radiotherapy + oral capecitabine) + total mesorecta excision (TME) surgery. The clinical efficacy of the treatments was evaluated through endoscopic, radiography, and tumor regression grade (TRG). In addition, expression level of RUNX3 and EZH2 was quantified *via* immunohistochemistry. The association of RUNX3 and EZH2 with clinicopathological characteristics of advanced tumors and efficacy of neoadjuvant therapy was explored. Logistic regression analysis was performed to identify predictors of efficacy of neoadjuvant chemoradiotherapy. Survival curve was used to evaluate the impact of RUNX3 and EZH2 on the prognosis of LARC patients.

**Results:**

A total of 80 patients diagnosed with LARC were enrolled in the study. Expression of RUNX3 was elevated in 25 (31.25%) patients, whereas expression of EZH2 was upregulated in 44 (55.00%) patients. Analysis of tumor regression identified 10 cases with TRG grade 0 (pathologic complete response, PCR), 24 cases with TRG grade 1, 35 cases with TRG grade 2, and 11 cases with TRG grade 3. Furthermore, 38 cases had significant down-staging, and 42 cases showed no significant down-staging as revealed by endoscopy and imaging. Patients with high expression of RUNX3 showed better tumor regression response and down-staging compared with those with low expression of RUNX3 (*P* < 0.001, *P* < 0.001). Moreover, patients with low EZH2 expression achieved TRG grade 0 and 1 response and down-staging effect compared with those with high expression of EZH2 (*P* < 0.001, *P* < 0.001). Logistic regression analysis showed that high expression of RUNX3, low expression of EZH2, and clinical N (cN) stage were good predictors of tumor regression response and down-staging. The 5-year disease free survival (DFS) and overall survival (OS) were 48.75 (39/80) and 58.75% (47/80), respectively. The 5-year DFS and OS of patients with high RUNX3 expression were significantly higher than low RUNX3 expression, whereas the 5-year DFS and OS of patients with high EZH2 expression were significantly lower than low EZH2 expression (*P* < 0.001). Univariate survival analysis showed that RUNX3 expression, EZH2 expression, cN, clinical T (cT), pathological T (pT) and pathological N (pN) were significantly correlated with the 5-year DFS and 5-year OS. Multivariate survival analysis showed that EZH2 expression and PN were good predictors of 5-year DFS and 5-year OS, whereas RUNX3 was a good predictor of 5-year DFS but not 5-year OS.

**Conclusions:**

Expression level of RUNX3 and EZH2 accurately predicts clinical efficacy of neoadjuvant chemoradiotherapy and the prognosis of LARC patients, suggesting that RUNX3 and EZH2 can be used as pivotal clinical predictors for LARC.

## 1 Introduction

Neoplasms remain the main killer worldwide (1, 2). Currently, the main diagnostic criteria of locally advanced rectal cancer (LARC) are based on distance to edge, transrectal intraperitoneal ultrasound (TIUS), chest and abdomen pelvic computed tomography (CT). For tumors of stage II/III, it is difficult to obtain enough circumferential margins and lymph node dissection to achieve R0 resection when performing direct surgery due to the anatomical location and pathological characteristics of the tumors. This results in a high postoperative local recurrence rate after surgery (3–6). Therefore, a “sandwich” treatment, comprising preoperative synchronous chemoradiotherapy (CRT) + total mesolectal resection (TME) + postoperative adjuvant chemotherapy, is generally applied in clinical practice to improve R0 resection rate and significantly reduce local recurrence rate (7, 8).

Currently, the clinical efficacy of CRT is mainly evaluated using endoscopic tools and imaging omics (rectal MRI + TIUS), which are influenced by experience of the surgeon and outcomes are susceptible to personal subjectivity. These assessment methods lack guidance from preoperative neoadjuvant therapy. Moreover, results of endoscope and imagological examination are not completely consistent with pathological regression of the tumor (9). Therefore, the latest American Society of Clinical Oncology (ASCO) guideline recommends that molecular biological indicators can be used to evaluate efficacy and prognosis of LARC (10, 11).

Previous studies report that abnormal expression of human related transcription factor-3 (RUNX3) and histone methyltransferase enhancer 2 (EZH2) contribute to the progression of colorectal cancer (12). EZH2 has been shown to regulate RUNX3 expression (13). Based on results reported in our previous work (14–16), we aimed to investigate whether RUNX3 and EZH2 can evaluate the clinical efficacy of neoadjuvant therapy and prognosis of LARC.

## 2 Methods

### 2.1 General Data

Clinical data of LARC patients admitted to Tianjin Medical University Cancer Institute/Hospital and First Affiliated Hospital of Hebei North University between January 1, 2015 and January 1, 2016 were retrospectively analyzed. All patients were diagnosed with rectal adenocarcinoma through pathological examination with a rectoscope. General information of the patients including age, sex, degree of differentiation, distance from the mass to the anal margin, clinical stage, surgical method, pathological type and pathological stage were recorded. Prior to treatment, TNM staging was determined through clinical examinations, including physical examination, carcinoembryonic antigen (CEA), chest, abdomen and pelvic enhanced CT, rectal magnetic resonance imaging (MRI), and TIUS examinations. TNM staging was determined following guidelines by the Staging Criteria of American Joint Committee on Cancer (AJCC) Eighth Edition. Patients with T3 or T4 or N+ and no distant metastasis (M0) were enrolled. This clinical study was approved by Tianjin Medical University Cancer Institute and Hospital and First Affiliated Hospital of Hebei North University ethics committees.

### 2.2 Preoperative Concurrent Chemoradiotherapy

Urine and feces were drained 1.5 hours before radiotherapy positioning to carry out long-course radiotherapy. The patient was requested to drink 500 mL water and 500 ml contrast medium. Plain scanning and enhanced CT localization were performed after thermoplastic film fixation under guidance of PHILIPS Bigbore 16 row CT. The patient was placed in supine position. Scanning range was from the lower edge of the liver to the upper 1/3 of the femur, and the layer thickness was 5 cm. The target area at Elekta Focal Station was outlined. Primary gross tumor volume (GTVp) was the primary lesion, including positive lymph nodes within the mesorectal and around the superior rectal artery. Gross tumor volume lymph nodes (GTVnd) was laterally metastatic lymph node, and CTV occurred on the mesorectal region, internal iliac region, obturator foramen, and presacral lymphatic drainage region. GTVp, GTVnd and clinical target volume (CTV) were expanded by 5mm to form PGTVp, PGTVnd, and PTV. Prescription dose was 95% PGTVp, 50.6Gy/PTV, 41.8Gy/22f, 95%PGTVnd and 50-60Gy/22f. Radiotherapy plan was performed using Elekta XIO planning system. Position correction was adjusted based on the original position CT machine. Intensity modulated radiotherapy was performed using the Elekta Syngery radiotherapy machine, once a day, 5 times a week.

All patients received concurrent chemotherapy and oral capecitabine (825 mg/m^2^, twice/d, 5 d/week, totally 5 week). After 2 weeks of rest after chemoradiotherapy, capecitabine was continued as a single drug for 2-3 cycles (1250 mg/m^2^, twice/d, continued for 2 weeks, the treatment was stopped for 1 week, and the second cycle was started).

### 2.3 Surgery

All patients were reassessed for down-staging status and tolerance after neoadjuvant therapy and before operation. Patients who met the criteria for surgery underwent radical resection for rectal cancer, including Dixon, Miles, and Hartmann. All surgeries were performed by the same surgical team in accordance with the TME principle.

### 2.4 Endoscopy

Indeed, the definitions of RECIST rules are different in various institutions, suggesting that RECIST rules cannot be used as an absolute evaluation standard. In this article, we use endoscopic method to visually evaluate the size of lesions after treatment. However, this method cannot be used as a method of evaluation. In this article, we use endoscopic method to visually evaluate the size of lesions after treatment, though the method of which failed to be used as a method of evaluation.

### 2.5 Imaging Omics Evaluation

The low rectal MRI staging criteria were used to evaluate the efficacy and the down-staging status based on the changes of tumor volume in MRI and transrectal intracavitary ultrasonography/section after neoadjuvant therapy.

#### 2.5.1 Imaging TNM Staging Diagnostic Criteria

In T1 stage, the tumor is limited to the mucosal layer or submucosa, and there is no obvious abnormal signal in the muscular layer. In T2 stage, the tumor invaded the muscle layer with continuous low signal band, and there was cord-like signal outside the wall, whereas the cord-like signal outside the wall were not adjacent to the outer edge of the tumor, and the signal was regular and natural. In T3 stage, the tumor broke through the low signal loop of the muscle layer, which was characterized by continuous interruption of the low signal band of the muscle layer, nodular convex tumor, blurred peri-intestinal fat space, and extramural burrs. In T4 stage, the tumor invaded the peritoneum and adjacent organs, exhibiting unclear boundary and adhesion with adjacent structures.

N stage: when the short diameter of lymph node is ≥ 1cm, it is considered as metastatic lymph node; When the short diameter of lymph node is 0.5 ~ 1cm, there are two situations: (1) when the boundary of lymph node is clear, the shape is regular, the internal signal is uniform or slightly uneven, the enhancement scan is uniform or slightly uneven, and the obvious enhancement belongs to benign lymph node; (2) malignant lymph nodes are those with unclear boundary, irregular shape, mild or obvious uneven internal signal, obvious uneven and mild to moderate enhancement on enhanced scan, or circular enhancement. When the short diameter of lymph node is ≤ 0.5cm, it is judged as benign lymph node.

#### 2.5.2 MRI Evaluation Method

Siemens 3.0 Tskyra MRI system and abdominal phased array coil are used for MRI scanning. Rectal scanning sequence includes sagittal T2WI fat suppression sequence, cross-sectional T2WI, high-resolution T2WI, diffusion weighted imaging (DWI), and enhancement sequence. The high-resolution T2WI is performed for oblique cross-section. The scanning plane is perpendicular to the long axis of the intestinal canal where the lesion is located. The scanning parameters are TR 4000 ms, TE 108 ms, FOV 18 cm, matrix 320 x 320, layer thickness 3 mm, no-interval scanning, 28 layers, reverse angle 150°, bandwidth 108 hz/pixel, no fat suppression, generalized self-calibration parallel acquisition mode, acceleration factor 3, acquisition time 250 s.

#### 2.5.3 MRI Tumor Regression Grade (mrTRG)

According to the Mandard pathology standard, mrTRG is divided into grade 1-5 according to the proportion of residual tumor tissue and fibrous tissue in the lesion after neoadjuvant chemoradiotherapy (NCRT). Grade 1: the tumor is completely relieved and there is no tumor residue on MRI image; Grade 2: severe treatment response, obvious low signal fibrous tissue in the diseased region, and the residual tumor tissue is not obvious; Grade 3: moderate treatment response, low signal fiber/mucus tissue and residual medium signal tumor tissue accounted for 50% of all image signals, respectively; Grade 4: mild treatment response, most of the diseased region are occupied by moderate tumor signals, and only a small amount of low signal fiber/mucus signals; Grade 5: there was no obvious therapeutic response, and the diseased region was still occupied by moderate tumor signals. mrTRG Grade 1-3 was defined as the group with good curative effects, and mrTRG grade 4-5 was defined as the group with poor curative effects.

#### 2.5.4 Ultrasonic Evaluation

Patients underwent endorectal ultrasonography (ERUS) examination before neoadjuvant therapy (ERUS1) and after NCRT therapy (ERUS2), using ultrasound equipment (BK Profocus 2202, Denmark), equipped with transrectal biplane probe 8848 and transrectal 360° circular scanning three-dimensional probe 8838/2052 (4-16 mHz); or ultrasound equipment (Yum mylab60) is equipped with transrectal biplane probe TRT 33 (4-13 mHz). The patient chosen the left lying position, bends his knees, was injected 50 mL coupling agent through the anus to fill the rectum, using the probe cover to protect the probe, and then inserts it through the anus from shallow to deep until the probe exceeds the upper edge of the tumor. The probe rotates clockwise for 360° circular scanning to determine the size, location, and best cross-sectional image of the diseased region. When using biplane probe 8848/TRT33, firstly, using linear array mode longitudinal scanning to collect the longitudinal section image of the longest diameter of the tumor. After careful observation, converting convex array mode transverse scanning to collect the transverse section image of the thickest diameter of the tumor. Using the three-dimensional imaging probe 8838/2502, after two-dimensional full observation, starting the three-dimensional volume automatic imaging, collect and store the image, and then intercepting the longitudinal section of the longest diameter and the cross section of the thickest diameter on the three-dimensional image. The longest diameter (longitudinal section measurement) and the thickest diameter (cross section measurement) of ERUS1 and ERUS 2 are measured by a non-examining doctor on the examination equipment respectively, and the average value is taken after three measurements. The length and thickness reduction rate are calculated. The calculation formula of the reduction rate is ΔERUSNCRT = (ERUS1-ERUS2)/ERUS1 x 100%”.

### 2.6 Pathological Assessment

Pathological examination was performed by two pathologists who were blinded to the patients’ clinical data. Postoperative TNM staging and down-staging status of tumors were evaluated based on the pathological results of the surgically resected specimen. Tumor response was determined using the tumor regression grade (TRG) system. TRG system is applied as follows: Grade 0; Complete tumor regression, a pathological complete response was achieved when only fibrous tissue or calcium salt deposits were seen, Grade 1; Moderate tumor regression, significant fibrosis accompanied by a small number of visible tumor cells or cell masses, Grade 2; Slight tumor regression, presence of a remnant tumor and a large amount of fibrotic interstitial filling, Grade 3; No tumor regression, extensive residual tumor, no or only a small amount of tumor cell necrosis. Patients were graded based on the TRG of surgical specimens. The response was defined as a good response (TRG 0-1) or a bad response (TRG2-3) (17).

### 2.7 Immunohistochemistry

Antibodies used to quantify RUNX3 expression (ab224641) and EZH2 expression (ab191080) were purchased from Abcam (Cambridge, UK). The expression of these proteins was determined using immunohistochemistry. Specimens obtained from preoperative biopsy tissue were cut into 5 μm sections. The sections were examined under a microscope. Five fields were evaluated, and the proportion of positive cells was counted, regardless of staining intensity. RUNX3 and EZH2 expression were divided into two groups: high expression group and low expression group. In the high expression group, at least 50% of the nuclei were positive whereas in the low expression group, the nucleus was less than 50% positive.

### 2.8 Follow up

Patients were closely followed up every 3 months for 2 years after treatment, and every 6 months thereafter. During follow up the patients underwent physical examination, serum carcinoma embryonic antigen (CEA), peripheral blood cell analysis, biochemistry tests, liver and kidney function tests, enhanced abdominal, and pelvic CT or MRI every 6 months. Electronic colonoscopy was performed 1 year later and then every 2 to 3 years. The median follow-up time was 60 months and the last follow-up time was December 31, 2020.

### 2.9 Statistical Analysis

All statistical analyses were carried out using SPSS 17.0 software. Chi-square test was used to analyze the association of RUNX3 and EZH2 expression with the clinical characteristics and treatment response of patients. Logistic regression analysis was performed to identify the predictors of sensitivity to preoperative chemoradiotherapy in patients with rectal cancer. Overall survival (OS) was defined as the duration from diagnosis to occurrence of death or withdrawal from follow-up. DFS was defined as the time from diagnosis to occurrence of recurrence or distant metastasis. Kaplan-Meier method was used to carry out univariate survival analysis. Cox proportional risk model was employed to perform multivariate survival analysis. *P* < 0.05 was considered as statistically significant.

## 3 Result

### 3.1 Association of RUNX3 and EZH2 Expression With Clinicopathological Characteristics of LARC

**Figure 1:**
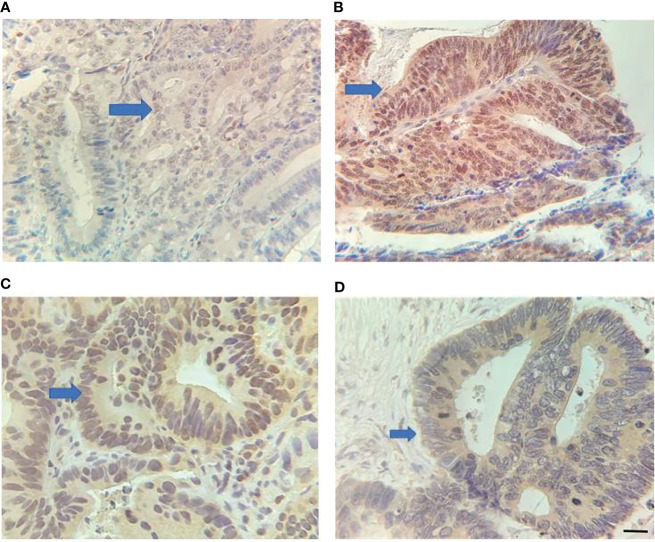
Expression of RUNX3 and EZH2 in LARC tissues. **(A)** Low RUNX3 expression; **(B)** high RUNX3 expression; **(C)** High EZH2 expression; **(D)** Low EZH2 expression. Scar bar = 25 μm.

A total of 80 LARC patients were enrolled in this study. Among them, 31 had clinical stage T3, 49 had T4, 38 had clinical stage N0, and 42 had clinical stage N+. In advanced CRC, RUNX3 was overexpressed in 31.25% (25/80) of patients and EZH2 was overexpressed in 55.00% (44/80) of patients. Expression levels of RUNX3 and EZH2 were correlated with CEA level, clinical T stage, and N stage, moreover, expression levels of RUNX3 were correlated with Ki-67 expression status. (**Table 1** and **Figure 1**).

**Table 1:** Expression of RUNX3 and EZH2 in LARC tissues and their relationship with clinicopathological factors.

Pathological Parameters	n	RUNX3		*χ^2^*	*P*	EZH2		*χ^2^*	*P*
		High Expression	Low Expression			High Expression	Low Expression		
Tumor Size				1.190	0.275			0.131	0.718
≥ 5 cm	36	9 (25.0%)	27 (75.0%)			19 (52.8%)	17 (47.2%)		
< 5 cm	44	16 (36.4%)	28 (63.6%)			25 (56.8%)	19 (43.2%)		
Differentiation Degree				4.762	0.092			0.204	0.903
High	21	10 (47.6%)	11 (52.4%)			12 (57.1%)	9 (42.9%)		
Medium	40	12 (30.0%)	28 (70.0%)			21 (52.5%)	19 (47.5%)		
Low	19	3 (15.8%)	16 (84.2%)			11 (57.9%)	8 (42.1%)		
Distance to the anal margin				0.093	0.760			0.349	0.555
≤ 5 cm	34	10 (29.4%)	24 (70.6%)			20 (55.00%)	14 (45.00%)		
> 5 cm	46	15 (32.6%)	31 (67.4%)			24 (52.00%)	22 (48.00%)		
Clinical T staging									
cT3	31	21 (67.7%)	10 (32.3%)	31.371	<0.001	6 (19.4%)	25 (80.6%)	25.983	<0.001
cT4	49	4 (8.2%)	45 (91.8%)			38 (77.6%)	11 (22.4%)		
Clinical N staging									
cN0	38	23 (60.5%)	15 (39.5%)	28.876	<0.001	7 (18.4%)	31 (81.6%)	39.130	<0.001
cN+	42	2 (4.8%)	40 (95.2%)			37 (88.1%)	5 (11.9%)		
CEA (ng/ml)				7.868	0.005			8.410	0.004
< 5	39	18 (46.2%)	21 (53.8%)			15 (38.5%)	24 (61.5%)		
≥ 5	41	7 (17.1%)	34 (82.92%)			29 (70.7%)	12 (29.3%)		
ki-67				3.902	0.048			0.115	0.734
Low expression	35	15 (42.9%)	20 (57.1%)			20 (57.1%)	15 (42.9%)		
High expression	45	10 (22.2%)	35 (77.8%)			24 (53.3%)	21 (46.7%)		

### 3.2 Assessment of LARC Treatment

**Figure 2:**
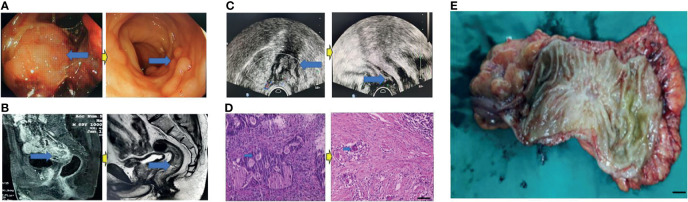
Surgical figures of PCR/TRG stage 0. **(C)** Transreetal ultrasound (Left graph: prior treatment; Right graph: Post treatment); **(B)** Rectal cancer MRI (Left graph: prior treatment; Right graph: Post treatment); **(A)** Endoscope (Left graph: prior treatment; Right graph: Post treatment); **(D)** HE staining (Left graph: prior treatment; Right graph: Post treatment), Scar bar = 50 μm; **(E)** Postoperative specimens, Scar bar = 1 cm.

**Figure 3:**
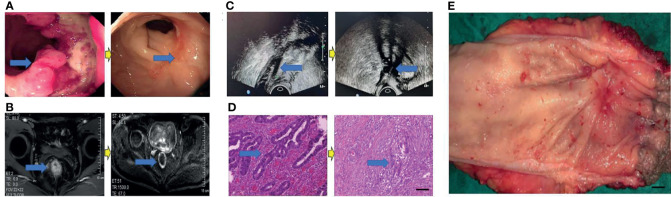
Surgical figures of TRG Grade 1. **(C)** Transreetal ultrasound (Left graph: prior treatment; Right graph: post treatment); **(B)** Rectal cancer MRI (Left graph: before treatment; Right graph: post treatment); **(A)** Endoscopy (Left graph: before treatment; Right graph: post treatment); **(D)** He staining (Left graph: before treatment; Right graph: post treatment), Scar bar = 50 μm; **(E)** Postoperative specimens, Scar bar = 1 cm.

**Figure 4:**
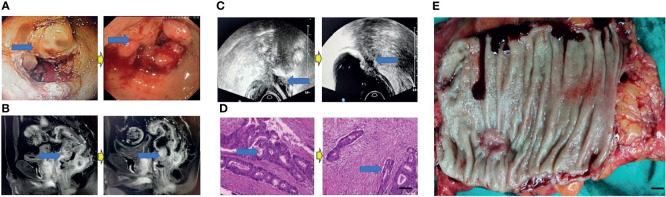
Surgical figures of TRG Grade 2. **(C)** Transreetal ultrasound (Left graph: before treatment; Right graph: post treatment); **(B)** Rectal cancer MRI (Left graph: before treatment; Right graph: post treatment); **(A)** Endoscopy (Left graph: before treatment; Right graph: post treatment); **(D)** He staining (Left graph: before treatment; Right graph: post treatment), Scar bar = 50 μm; **(E)** Postoperative specimens, Scar bar = 1 cm.

**Figure 5:**
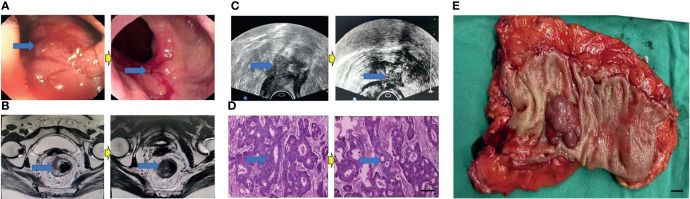
Surgical figures of TRG Grade 3. **(C)** Transreetal ultrasound (Left graph: before treatment; Right graph: post treatment); **(B)** Rectal cancer MRI (Left graph: before treatment; Right graph: post treatment); **(A)** Endoscopy (Left graph: before treatment; Right graph: post treatment); **(D)** He staining (Left graph: before treatment; Right graph: post treatment), Scar bar = 50 μm. **(E)** Postoperative specimens, Scar bar = 1 cm.

Eighty patients successfully underwent examinations after treatment. Analysis of tumor regression showed 10 cases with TRG grade 0 (PCR) (12.50%), 24 cases with TRG grade 1 (30.00%), 35 cases with TRG grade 2 (43.75%), and 11 cases with TRG grade 3 (13.75%). Endoscopic evaluation showed that 41 cases (51.25%) were effective, 38 cases (47.50%) had significant down-staging, and 42 cases (52.50%) had no significant down-staging (**Figures 2**–**5**).

### 3.3 Relationship Between Expression of RUNX3 and EZH2 and Other Clinical Factors and Response to Neoadjuvant Chemoradiotherapy

Patients with high expression of RUNX3 were more sensitive to neoadjuvant chemoradiotherapy compared with those with low expression of RUNX3. On the contrary, patients with low expression of EZH2 were more sensitive to neoadjuvant chemoradiotherapy compared with those with high expression of EZH2. 22 out of the 25 patients with high expression of RUNX3 achieved TRG grade 0/1, whereas only 12 of the 55 patients with low expression of RUNX3 achieved good tumor regression after treatment (*P* < 0.001). 29out of the 36 patients with low expression of EZH2 achieved good tumor regression, whereas only 5 of the 44 patients with high expression of EZH2 achieved good tumor regression (*P* < 0.001). Analysis showed that 23 out of the 25 patients with high expression of RUNX3 presented with tumor decline, whereas only 15 of the 55 patients with low expression of RUNX3 presented with tumor decline (*P* < 0.001). Out of the 36 patients with low expression of EZH2, 30 presented with tumor decline, whereas 8 of the 44 patients with high expression of EZH2 presented with tumor decline *(P* < 0.001). CEA < 5 ng/ml and CN0 were associated with good tumor regression and down-staging (*P* = 0.001 P = 0.014; *P* < 0.001, *P* < 0.001). Patients with cT3 were more likely to achieve the desired tumor regression response compared with patients with cT4 (**Table 2**).

**Table 2:** Relationship between clinicopathological characters and neoadjuvant therapy efficacy for LARC.

Pathological Parameters	n	Tumor Regression		*χ^2^*	*P*	Down-Staging		*χ^2^*	*P*
		TRG 0/1	TRG 2-3			Yes	No		
Tumor Size				0.349	0.555			0.245	0.621
≥ 5 cm	36	14 (38.9%)	22 (61.1%)			16 (44.4%)	20 (55.6%)		
< 5 cm	44	20 (45.5%)	24 (54.5%)			22 (50.0%)	22 (50.0%)		
Differentiation Degree				1.766	0.413			1.184	0.553
High	21	11 (52.4%)	10 (47.6%)			12 (57.1%)	9 (42.9%)		
Medium	40	17 (42.5%)	23 (57.5%)			17 (42.5%)	23 (57.5%)		
Low	19	6 (31.6%)	13 (68.4%)			9 (47.4%)	10 (52.6%)		
Distance to the anal margin				0.042	0.837			0.948	0.330
≤ 5 cm	34	14 (41.2%)	20 (58.8%)			14 (41.2%)	20 (58.8%)		
> 5 cm	46	20 (43.5%)	26 (56.5%)			24 (52.2%)	22 (47.8%)		
Clinical T staging				16.784	<0.001			14.462	<0.001
cT3	31	22 (71.0%)	9 (29.0%)			23 (74.2%)	8 (25.8%)		
cT4	49	12 (24.5%)	37 (75.5%)			15 (30.6%)	34 (69.4%)		
Clinical N staging				33.869	<0.001			33.709	<0.001
cN0	38	29 (76.3%)	9 (23.7%)			31 (81.6%)	7 (18.4%)		
cN+	42	5 (11.9%)	37 (88.1%)			7 (16.7%)	35 (83.3%)		
CEA (ng/ml)				11.287	0.001			6.014	0.014
< 5	39	24 (61.5%)	15 (38.5%)			24 (61.5%)	15 (38.5%)		
≥ 5	41	10 (24.4%)	31 (75.6%)			14 (34.1%)	27 (65.9%)		
RUNX3				30.806	<0.001			28.876	<0.001
High expression	25	22 (88.0%)	3 (12.0%)			23 (92.0%)	2 (8.0%)		
Low expression	55	12 (21.8%)	43 (78.2%)			15 (27.3%)	40 (72.7%)		
EZH2				38.790	<0.001			33.702	<0.001
High expression	44	5 (11.4%)	39 (88.6%)			8 (18.2%)	36 (81.8%)		
Low expression	36	29 (80.6%)	7 (19.4%)			30 (83.3%)	6 (16.7%)		

### 3.4 Predictors of the Efficacy of Neoadjuvant Chemoradiotherapy in Patients With LARC

Multiple logistic regression analysis showed that high expression of RUNX3 and low expression of EZH2 were significantly associated with good tumor regression (TRG grade 0/1) (*P* = 0.021, *P* = 0.016) and tumor down-staging (*P* = 0.014, *P* = 0.043). In addition, CN was found to be a predictor of tumor regression response (*P* = 0.010) and tumor decline stage (*P* = 0.008, **Table 3**).

**Table 3:** Multiple logistic regression analysis of the predictors of efficacy of neoadjuvant therapy for LARC.

Factors	OR	95%CI	*P*
TRG 0/1			
CEA	4.841	0.945-24.787	0.058
cT	0.053	0.003-1.100	0.058
cN	25.003	2.170-288.138	0.010
RUNX3 expression	0.105	0.015-0.716	0.021
EZH2 expression	9.559	1.535-59.521	0.016
Tumor Down-staging			
CEA	1.613	0.377-6.897	0.519
cT	0.061	0.004-1.077	0.056
cN	26.906	2.363-306.333	0.008
RUNX3 expression	0.090	0.013-0.613	0.014
EZH2 expression	5.476	1.059-28.324	0.043

### 3.5 Survival Follow-up

**Figure 6:**
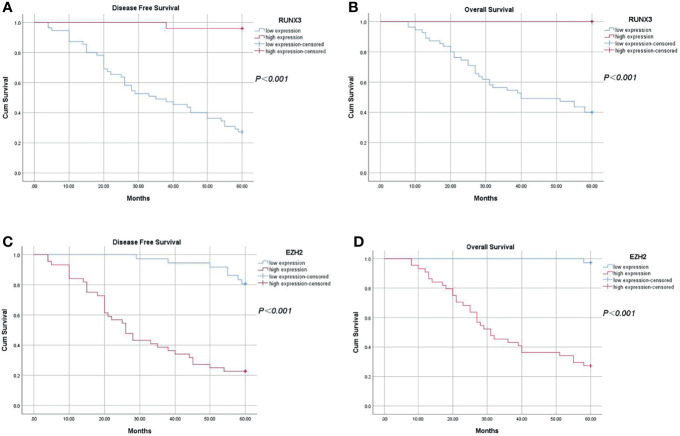
Kaplan-Meier analysis of the relationship between expression of RUNX3 and EZH2 and 5-year disease-free survival and overall survival in LARC patients. **(A)** 5-year disease-free survival of patients with high expression of RUNX3 was significantly higher compared with that of patients with low expression of RUNX3 (*P* < 0.05); **(B)** Overall survival of patients with high expression of RUNX3 was significantly higher compared with that of patients with low expression (*P* < 0.05). **(C)** 5-year disease-free survival of patients with high expression of EZH2 was significantly lower compared with that of patients with low expression of EZH2 (*P* < 0.05). **(D)** Overall survival time of patients with high expression of EZH2 was significantly lower compared with that of patients with low expression of EZH2 (*P* < 0.05).

Complete follow-up data were obtained for all 80 patients, with a median follow-up time of 60 months. Analysis showed that the 5-year DFS was 48.75% (39/80) and 5-year OS was 58.75% (47/80). The 5-year DFS and 5-year OS of patients with high expression of RUNX3 were 96.00% (24/25) and 100.00% (25/25), respectively. On the other hand, the 5-year DFS and 5-year OS of patients with low expression of RUNX3 were 27.30% (15/55) and 40.00% (22/55), respectively (*P* < 0.001). The 5-year DFS and 5-year OS of patients with high expression of EZH2 were 22.70% (10/44) and 35.30% (12/44), respectively. The 5-year DFS and 5-year OS of patients with low expression of EZH2 were 80.60% (29/36) and 97.20% (35/36), respectively (*P* < 0.001, **Figure 6**).

### 3.6 Risk Factor Survival Analysis

Univariate survival analysis showed that expression of RUNX3 and EZH2, cN, cT, pT, and pN were significantly correlated with 5-year DFS and 5-year OS. In addition, results of multivariate analysis demonstrated that EZH2 expression and pN were predictors of 5-year DFS and 5-year OS, whereas RUNX3 was a predictor of 5-year DFS, but not a predictor of the 5-year OS (**Tables 4**, **5**).

**Table 4:** Univariate survival analysis of LARC.

Factors	n	5-year DFS	*x^2^*	*P*	5-yesar OS	*x^2^*	*P*
Tumor Size			1.213	0.271		1.693	0.193
≥ 5 cm	36	20 (55.6%)			24 (66.7%)		
< 5 cm	44	19 (43.2%)			23 (52.3%)		
Differentiation Degree			0.572	0.751		1.111	0.574
High	21	10 (47.6%)			11 (52.4%)		
Medium	40	21 (52.5%)			23 (57.5%)		
Low	19	8 (42.1%)			13 (68.4%)		
Distance to the anal margin			1.357	0.244		0.823	0.364
≤ 5 cm	34	14 (41.2%)			18 (52.9%)		
> 5 cm	46	25 (54.3%)			29 (63.0%)		
Clinical T staging			20.608	<0.001		13.179	<0.001
cT3	31	25 (80.6%)			26 (83.9%)		
cT4	49	14 (28.6%)			21 (42.9%)		
Pathological T staging			16.540	<0.001		13.316	<0.001
pT0~2	19	17 (89.5%)			18 (94.7%)		
pT3~4	61	22 (36.1%)			29 (47.5%)		
Clinical N staging			31.223	<0.001		28.193	<0.001
cN0	38	31 (81.6%)			34 (89.5%)		
cN+	42	8 (19%)			13 (31.0%)		
Pathological N staging			25.379	<0.001		27.600	<0.001
pN0	47	34 (72.3%)			39 (83.0%)		
pN^+^	33	5 (15.2%)			8 (24.2%)		
CEA (ng/ml)			1.787	0.181		3.449	0.063
< 5	39	22 (56.4%)			27 (69.2%)		
≥ 5	41	17 (41.5%)			20 (48.8%)		
RUNX3			32.494	<0.001		25.532	<0.001
High expression	25	24 (96.0%)			25 (100.0%)		
Low expression	55	15 (27.30%)			22 (40.00%)		
EZH2			26.502	<0.001		30.397	<0.001
High expression	44	10 (22.70%)			12 (35.30%)		
Low expression	36	29 (80.60%)			35 (97.20%)		

**Table 5:** Multivariate survival analysis of LARC.

Factors	Regression Coefficient	Standard Error	Statistic	*P*	Risk Ratio	*95% CI*
5-year DFS						
RUNX3	-2.571	1.146	5.036	0.025	0.076	0.008-0.722
EZH2	0.945	0.456	4.290	0.038	2.573	1.052-6.291
pT	-0.011	0.800	0.000	0.989	0.989	0.206-4.747
pN	0.986	0.364	7.318	0.007	2.680	1.312-5.474
5-year OS						
RUNX3	-11.091	144.245	0.006	0.939	0.000	–
EZH2	2.632	1.029	6.550	0.010	13.906	1.852-104.398
pT	0.013	1.025	0.000	0.990	1.013	0.136-7.558
pN	0.902	0.421	4.598	0.032	0.465	1.081-5.621

## 4 Discussion

### 4.1 Main Finding

In this study, 80 LARC patients were enrolled and accompanied by a median 60-months follow-up. Expression of RUNX3 and EZH2 can accurately evaluate treatment efficacy of neoadjuvant chemoradiotherapy and effective predictors of the prognosis of LARC patients. Therefore, RUNX3 and EZH2 have significant clinical implications.

### 4.2 Interpretation

The spread of tumors through intestinal wall and extraserosal as well as mesangial lymph nodes metastasis are important clinicopathological indicators of the prognosis of LARC patients. The sandwich treatment mode can decrease local recurrence rate and increase survival rate of LARC patients more effectively than simple operation and postoperative adjuvant chemotherapy. Preoperative neoadjuvant therapy is superior to traditional postoperative radiotherapy and chemotherapy in terms of local control rate and reducing toxic reactions (18). Preoperative chemoradiotherapy reduces the depth of tumor invasion in the intestinal wall by killing tumor cells, and completely clears tumor cells to achieve pathological PCR. Previous studies have shown that approximately 15%-40% of LARC patients achieve PCR after neoadjuvant therapy (19). In China, several research centers have shown that the PCR rate of LARC patients receiving neoadjuvant therapy before surgery is approximately 20%. In addition, 20% to 30% of these patients achieve significant or moderate regression. Although neoadjuvant therapy has a high overall effective rate, some patients show non-regression or tumor progression after treatment (implying that the tumor is not sensitive to radiotherapy or chemotherapy) (20). Therefore, a comprehensive and accurate evaluation system should be developed for accurate evaluation of efficacy of neoadjuvant therapy.

Traditional endoscope only reveals the size of tumor and proportion of the annulus lumen. It also analyzes tumor shrinkage by comparing with pre-treatment images, which allows evaluation of the effect of neoadjuvant therapy. TIUS has been used to explore the efficacy of neoadjuvant therapy in LARC patients in recent years. Multimodal ultrasonomics, such as conventional transrectal ultrasound, elastography, shear wave, contrast-enhanced ultrasound, and other modes, can be used to measure tumor length, thickness reduction rate, and blood flow before and after treatment. Thus, they can be employed to assess clinical efficacy of treatments. However, these methods are limited by the shape and location of rectal tumors. Therefore, better evaluation methods are needed. Before TIUS, rectal MRI was used to evaluate the efficacy of neoadjuvant therapy in LARC patients. MRI tumor regression grade (mrTRG) is a valuable imaging indicator that reflect the effectiveness and ineffectiveness of rectal cancer treatments (21). In recent years, FDF-PET, DWI, and DCE-MRI have been used to complement anatomy-based high-resolution MRI efficacy evaluation methods by providing information on tumor cell metabolism, cell density, and blood perfusion. However, rectal MRI is not sufficiently accurate, as it is affected by objective factors such as tumor location and subjective factors of the viewer.

RUNX3 is a tumor suppressor gene that is located on human chromosome 1p36 and has a size of 67kb. RUNX3 protein is a heterodimer containing 415 amino acid residues. Silencing and inactivation of this gene promotes occurrence of cancer. RUNX3 inhibits growth of tumor cells by regulating the transcriptional growth factor β (TGF-β) and Wnt signaling pathways (13). EZH2 is a member of the newly discovered PcG gene family. EZH2 is involved in the regulation of cell cycle, and its high expression can accelerate entry of cells into the S phase, and promote cell proliferation (22–24). Lian et al. reported that EZH2 may regulate proliferation and apoptosis of laryngeal cancer cells by targeting expression of RUNX3 through Wnt/β-catenin signaling pathway (25).

In this study, we explored, for the first time, expression of RUNX3 and EZH2 proteins in LARC tissues. We found that RUNX3 was highly expressed in 31.25% of LARC patients whereas EZH2 was highly expressed in 55.00% of the patients. Expression status of RUNX3 and EZH2 was correlated with CEA level, clinical T stage, and N stage, whereas expression status of RUNX3 was correlated with Ki-67 expression status. Further analysis revealed that patients with high expression level of RUNX3 responded well to chemoradiotherapy compared with those with low expression of RUNX3, hence showed significant regression and down-staging. On the contrary, low expression of EZH2 was correlated with better response to chemoradiotherapy. This implies that the expression status of the two genes, and CN staging, can be used as independent indicators of efficacy of neoadjuvant therapy in LARC patients. Sensitivity of the body to chemoradiotherapy can be affected by a number of factors, including cell cycle arrest, DNA damage repair, and apoptosis. Cells at different stages of cell cycle have different sensitivities to radiation and drugs. For instance, G2/M phase is highly sensitive to therapy, whereas S phase has low sensitivity to therapy. In the classical RUNX3/TGF-β pathway, RUNX3 binds specifically to Smad and activates P21 promoter. P21 promoter enhances transcription of pro-apoptotic gene BIML and expression of cyclo-dependent kinase inhibitor p21WAFI in tumor cells, causing cell arrest at the G1 phase and inhibition of cell proliferation (26). EZH2 is a cell cycle regulator, and its overexpression shortens the G1 phase and causes accumulation of cells in the S phase, resulting in a significant increase in the number of cells in the G2/M phase.

In addition, the findings of this study show that RUNX3 and EZH2 are molecular biological indicators of poor prognosis in LARC. Furthermore, univariate, and multivariate analyses showed that RUNX3 and EZH2 expression levels are effective predictors of the survival.

### 4.3 Limitations

This study has some limitations. Firstly, the number of patients with LARC included in the study were relatively small. Therefore, further multi-center clinical case studies are needed to validate the findings of this study. Secondly, this article is a retrospective study and further prospective studies are expected. Thirdly, with the development of artificial intelligence technology, artificial intelligence technology can be introduced into evaluation of neoadjuvant therapy for LARC patients in the future, to evaluate the curative effect more accurately and improve quality of life of patients.

### 4.4 Conclusion

The molecular biological indicators of efficacy of neoadjuvant therapy in patients with LARC were explored using various methods including endoscopy, imageology (transrectal ultrasound, rectal MRI), and pathology.

In summary, the efficacy and prognostic value of RUNX3 and EZH2 in LARC patients receiving neoadjuvant concurrent chemoradiotherapy was investigated in this study. The results of this study have significant clinical implications. However, this study also had some shortcomings, such as enrolment of relatively few patients with LARC and high tumor heterogeneity among the enrolled patients. Therefore, further clinical studies should be performed to validate the present findings.

## Data Availability Statement

The original contributions presented in the study are included in the article/supplementary material. Further inquiries can be directed to the corresponding author.

## Ethics Statement

The studies involving human participants were reviewed and approved by Tianjin Medical University Nankai Hospital and Hebei North University. The patients/participants provided their written informed consent to participate in this study. Written informed consent was obtained from the individual(s) for the publication of any potentially identifiable images or data included in this article.

## Author Contributions

LW participated in Data Visualization, Conceptualization, Writing - Original Draft, and Formal analysis. XW participated in Formal analysis and Data Curation. WX participated in Conceptualization, Resources, Review & Editing, Supervision, Project administration, and Funding acquisition. LG participated in Methodology and Software. XMW participated in Conceptualization, Investigation, Review & Editing. T L participated in Writing, Review & Editing. All authors contributed to the article and approved the submitted version.

## Funding

This study was funded by Project of The National Cancer Center Cancer Research (NCC2017A19) and Project for High-level Talents of Hebei Province (No.A202101062).

## Conflict of Interest

The authors declare that the research was conducted in the absence of any commercial or financial relationships that could be construed as a potential conflict of interest.

## Publisher’s Note

All claims expressed in this article are solely those of the authors and do not necessarily represent those of their affiliated organizations, or those of the publisher, the editors and the reviewers. Any product that may be evaluated in this article, or claim that may be made by its manufacturer, is not guaranteed or endorsed by the publisher.

## Abbreviations

RUNX3, Runt-Related Transcription Factor 3; EZH2, enhancer of zeste homolog 2; LARC, locally advanced rectal cancer; TME, total mesorecta excision; TRG, tumor regression grade; DFS, disease free survival; OS, overall survival; TIUS, transrectal intraperitoneal ultrasound; cN, clinical N; cT, clinical T; pT, pathological T; pN, pathological N; CT, computed tomography; CRT, synchronous chemoradiotherapy; ASCO, American Society of Clinical Oncology; CEA, carcinoembryonic antigen; MRI, rectal magnetic resonance imaging; AJCC, American Joint Committee on Cancer; GTVnd, Gross tumor volume lymph nodes; GTVp, primary gross tumor volume; CTV, clinical target volume; RECIST, Response Evaluation Criteria in Solid Tumors; DWI, diffusion weighted imaging; mrTRG, MRI tumor regression grade; NCRT, neoadjuvant chemoradiotherapy; ERUS, endorectal ultrasonography; TRG, tumor regression grade; CEA, carcinoma embryonic antigen.
